# Artificial Intelligence in the Heart of Medicine: A Systematic Approach to Transforming Arrhythmia Care with Intelligent Systems

**DOI:** 10.2174/011573403X334095241205041550

**Published:** 2025-02-03

**Authors:** Adel Khalifa Sultan Hamad, Jassim Haji

**Affiliations:** 1 Department of Electrophysiology, Mohammed Bin Khalifa Bin Salman Al Khalifa Cardiac Centre, Awali, Kingdom of Bahrain;; 2 International Group of Artificial Intelligence, Manama, Kingdom of Bahrain

**Keywords:** Artificial intelligence, arrhythmias, cardiology, cardiovascular disease, heart failure, machine learning

## Abstract

**Background:**

At a critical juncture in the ongoing fight against cardiovascular disease (CVD), healthcare professionals are striving for more informed and expedited decision-making. Artificial intelligence (AI) promises to be a guiding light in this endeavor. The diagnosis of coronary artery disease has now become non-invasive and convenient, while wearable devices excel at promptly detecting life-threatening arrhythmias and treatments for heart failure.

**Objective:**

This study aimed to highlight the applications of AI in cardiology with a particular focus on arrhythmias and its potential impact on healthcare for all through careful implementation and constant research efforts.

**Methods:**

An extensive search strategy was implemented. The search was conducted in renowned electronic medical databases, including Medline, PubMed, Cochrane Library, and Google Scholar. Artificial Intelligence, cardiovascular diseases, arrhythmias, machine learning, and convolutional neural networks in cardiology were used as keywords for the search strategy.

**Results:**

A total of 6876 records were retrieved from different electronic databases. Duplicates (N = 1356) were removed, resulting in 5520 records for screening. Based on predefined inclusion and exclusion criteria, 4683 articles were excluded. Following the full-text screening of the remaining 837 articles, a further 637 were excluded. Ultimately, 200 studies were included in this review.

**Conclusion:**

AI represents not just a development but a cutting-edge force propelling the next evolution of cardiology. With its capacity to make precise predictions, facilitate non-invasive diagnosis, and personalize therapies, AI holds the potential to save lives and enhance healthcare quality on a global scale.

## 1 INTRODUCTION

Artificial intelligence (AI), in general, is a field within engineering that applies novel concepts to address intricate problems [1]. It is a rapidly evolving multidisciplinary domain that integrates computer science, statistics, psychology, neuroscience, material science, mechanical engineering, and computer hardware design to develop algorithms that seek to emulate human intuition, decision-making, and object recognition [2]. AI is a broader term for technologies or systems capable of exhibiting human-like intelligence. Machine learning (ML), on the other hand, is a subset of AI and specifically pertains to the ability of machines to learn from data, improve their performance with experience, and generate forecasts [3]. AI methodologies are increasingly being adopted into every face of patient care and opening doors to minimally invasive or non-invasive treatment methods [4]. AI is undergoing remarkable advancements and significantly improving the capability and performance of diagnostic systems. Moreover, it has the potential to optimize treatment procedures, thus, resulting in increased therapeutic efficiency, patient satisfaction, and lower expenses [5, 6]. The performance of AI technology depends on the appropriate utilization of relevant data and its quality, and it is essential to focus on solutions for more extensive and adaptable data sharing while reducing the necessity for human annotation, such as semi-supervised and transfer learning methods [7].

AI has been employed in medical imaging for over two decades, contributing to the advancement of computer-aided diagnosis and therapy solutions aimed at supporting healthcare providers [8]. Digital technologies serve as monitoring tools to produce substantial data in cardiology [9]. The utilization of ML in cardiovascular research has experienced exponential growth in recent years, with several pioneering applications already being used in routine clinical practice [10]. AI techniques, including ML, deep learning (DL), and cognitive computing, can play a pivotal role in the early detection and diagnosis of cardiovascular disease (CVD), along with forecasting outcomes and evaluating prognosis [11]. The widespread access to and consumer appeal of these devices has resulted in a wealth of data that is widely available to the healthcare sector. This data encompasses optical sensor-derived photoplethysmography (PPG) signals, electrocardiograms (ECGs), and accelerometers, which enable the measurement of physical activity [12]. One of the initial applications of AI in echocardiography was the automated quantification of left ventricular (LV) volume and function, which expedited the assessment process significantly. Computerized ECG interpretation models have enhanced the physician’s ability to read ECGs more rapidly. These models are widely used across all hospitals, but they can be prone to inaccuracies, and excessive reliance on them may lead to incorrect treatment decisions and unnecessary testing [13]. The coronary artery calcium (CAC) score has advanced the assessment of coronary atherosclerosis identification and risk stratification. Following the acquisition of images, AI is employed to verify that all the acquired images adhere to prescribed imaging standards for subsequent processing and analysis. Myocardial perfusion imaging (MPI) through single-photon emission computed tomography (SPECT) and positron emission tomography (PET) plays a pivotal role in the diagnosis and management of coronary artery disease (CAD) [7]. Noseworthy *et al.*, in a non-randomized interventional trial, applied an AI algorithm to the ECGs to divide patients into high-risk or low-risk groups. Compared with usual care, AI-guided screening was associated with increased detection of atrial fibrillation (high-risk group: 3.6% [95% CI 2.3-5.^4^] with usual care vs 10·6% [8.3-13.^2^] with AI-guided screening, *p*<0·0001; low-risk group: 0.9% *vs* 2.4%, p=0.12) over a median follow-up of 9.9 months (IQR 7.1-11.0) [14]. Shakibfar *et al.* investigated the possibility of predicting electrical storm (ES) based on daily stored implantable cardioverter-defibrillators ICD summaries using two models [logistic regression (LR) and random forest (RF)] on a large dataset. A modern ML model (RF) performed better than a standard LR model, with area under the curve (AUC) of 0.80 *vs* 0.75, respectively [15].

This review addresses AI in cardiology to improve heart disease detection, treatment, and management, with particular emphasis on arrhythmias. Specifically, it explores AI applications such as ML and convolutional neural networks (CNNs) in automated ECG analysis, AI-based cardiovascular risk stratification, and decision support systems for personalized treatment. The review covers emerging trends in AI research, clinical trials demonstrating improved outcomes, and the ethical and regulatory challenges associated with integrating AI into healthcare. By assessing current advancements and future directions, this review aims to highlight the impact of intelligent systems on arrhythmia management, contributing to more precise and efficient cardiovascular care.

## 2 METHODOLOGY

### 2.1 Searching Strategy

An extensive search strategy was implemented to gather comprehensive insights into novel treatment strategies for AI in CVD. The search was conducted in renowned medical databases, including Medline, PubMed, and the Cochrane Library. In addition, manual searches were performed on Google Scholar, and references from established publications were reviewed.

Keywords employed in the literature search included “Artificial Intelligence and Cardiovascular Diseases”, “Artificial Intelligence and Arrhythmias”, “Automated ECG Analysis with Machine Learning”, “AI-based Cardiovascular Risk Assessment”, “Convolutional Neural Networks in Cardiology”.

### 2.2 Study Selection and Data Collection (Inclusion Exclusion Criteria)

The selection of studies for this exploration was meticulous and rigorous. Initially, articles were considered based on their titles and abstracts. Subsequently, a thorough assessment of the full-text articles was conducted. Articles specifically addressed novel treatment strategies for AI in CVD that were published in English during 2018-2024 were included.

For data extraction, relevant information was gathered, such as the author’s name, year of publication, study design, study duration, sample size, and key findings from each study. The preferred reporting items for systematic reviews and meta-analyses (PRISMA) scoping review checklist were the guiding framework for data collection and reporting.

A comprehensive literature search was conducted utilizing different electronic databases: Cochrane Library with Full Text, Web of Science, PubMed, Google Scholar, Elsevier, Springer, MDPI, and Medline with full text. A total of 6876 records were retrieved from Cochrane Library with Full Text (N = 958), Web of Science (N = 945), PubMed (N=1795), Google Scholar (N=2095), Elsevier (N=467), Springer (N=336), MDPI (N=148), and Medline with full text (N = 132). No further records were identified through additional sources. Articles were exported to reference management software in Endnote for subsequent analysis. Duplicates (N = 1356) were removed, resulting in a pool of 5520 records for title and abstract screening. Based on predefined inclusion and exclusion criteria, 4683 articles were excluded due to thematic irrelevance. Following the full-text screening of the remaining 837 articles, a further 637 were excluded due to editorials, letters, short communications, and retractions. Ultimately, 200 studies were eligible for included based on this review, and the detailed selection process is depicted in Fig. (**1**).

## 3 RESULTS

### 3.1 Importance of AI in Cardiology

Cardiovascular imaging stands out as one of AI's most dynamic clinical applications due to the unique challenges posed by processing images of a constantly beating organ [8]. AI applications are currently being deployed to expedite image acquisition and reduce reconstruction time in cardiac MRI, automate disease classification in echocardiography, and enhance traditional risk prediction models based on coronary CT angiography features [16]. Implementing AI in cardiovascular medicine aims to improve data collection and analysis precision, leading to more accurate disease diagnosis, earlier disease detection, and better-predicting outcomes [17]. The development of mobile and wearable technologies with AI is swiftly transforming the disease detection and diagnostic landscape in cardiac electrophysiology [18]. Wearable devices are user-friendly and allow for uninterrupted monitoring and real-time individual analysis of ECG signals. The randomized controlled trial (RCT) results focused on AF screening, involving twice-weekly single-lead ECGs in ambulant patients aged 65 and over at high risk of stroke over 12 months, significantly improved AF detection compared to routine monitoring [19]. Another RCT assessed the occurrence of recurrent AF or atrial flutter through daily ECG self-recordings and the time to treatment of the recurring arrhythmia in patients undergoing catheter radiofrequency ablation or direct current cardioversion for AF or atrial flutter [20]. AI technologies play a role in analyzing and interpreting ECG data, predicting the likelihood of recurrent arrhythmias, or optimizing the timing of interventions based on individual patient characteristics [18, 21, 22]. Most of the functions in pacemakers and defibrillators rely on rule-based algorithms. For instance, the rate response feature in pacemakers, which allows the device to adjust the pacing rates based on sensor input, and the tachycardia detection and delivery of appropriate therapy by an implantable defibrillator are examples of rule-based decision-making [4]. Particularly in complex aortic cases and arrhythmic procedures, AI can leverage three-dimensional (3D) image data to achieve precise tissue access. The AI-based navigation systems aid in accurately guiding catheters and optimizing intervention plans [23]. Introducing minimally invasive approaches and surgical robots in cardiac surgery promises swifter recovery and reduced postoperative complications and mortality [24].

AI has demonstrated the ability to rectify existing medication errors, making it a valuable ‘assisting resource’ that clinicians can rely on and incorporate into their daily clinical practice. Typically, when heart problems are suspected, the first-line investigation involves an ECG, after which the diagnosis is narrowed down. If heart failure (HF) is suspected, physicians often investigate cardiac biomarkers [25]. An AI-clinical decision support system, developed by combining expert knowledge with an ML approach for diagnosing HF with varying ejection fractions (EF) (reduced, mildly reduced, and preserved), has shown improved diagnostic accuracy compared to relying solely on either expert-driven or ML approaches [26]. AI is being used to develop a more sophisticated and intelligent clinical decision support system (CDSS) that can provide more personalized and actionable decision support to clinicians in cardiology. By analyzing a patient’s unique clinical profile, AI-driven CDSS can assist clinicians in making more accurate diagnoses, selecting optimal treatments, and predicting patient outcomes. This decision-making enhancement improves the quality of care and increases the efficiency of healthcare delivery, ultimately leading to better patient outcomes. Fig. (**2**) represents the flow of CDSS.

Digital online image analysis and visualization methods have been devised to aid physicians during percutaneous coronary interventions (PCI). Stent visibility can be suboptimal using X-ray fluoroscopy, prompting the development of digital image processing techniques for stent enhancement. A comprehensive review of stent enhancement highlights its utility in evaluating stent strut damage, stent overlap, stent failure, aortic-ostial lesions, and bifurcations [27]. AI possesses the capability for real-time continuous monitoring (whether in a home or healthcare setting) with integrated care pathways for escalation and interventions when necessary, ultimately empowering patients in self-management, thereby enhancing clinical outcomes [28].

Valvular heart disease (VHD) typically presents as a chronic and progressively worsening condition characterized by a gradual and insidious onset that is clinically undetectable for many years until it is diagnosed. Following diagnosis might be required, which may lead to a cure, although it often requires continuous long-term monitoring [29]. Recent advancements in AI technology have shown promise in leveraging ECG data for certain cardiac conditions [22]. The AI ECG was designed using a convolutional neural network (CNN), with the primary aim of identifying patients afflicted with moderate to severe aortic stenosis (AS), and holds the potential to function as a powerful screening tool for AS within the broader community [30]. The CNN is trained on large datasets containing ECG recordings from individuals with confirmed cases of AS and those without the condition. This training process enables the AI to learn and discern specific features associated with the electrocardiographic manifestations of AS [31]. Incorporating AI into the analysis of extensive datasets through bioinformatics and systems biology tools holds the potential to accelerate drug target discovery. Therapeutic targets validated in experimental models of various diseases may be assessed clinically by employing advanced imaging techniques to identify effective treatment therapies for cardiovascular calcification [32].

CAD stands as a significant global contributor to morbidity and mortality worldwide, necessitating effective management that heavily relies on cardiac imaging to identify individuals who may require further treatment. Stress echocardiography (SE) is one of the most frequently employed techniques for non-invasive CAD assessment, mainly due to its cost-effectiveness, absence of ionizing radiation, and high patient tolerability [33]. Coronary artery calcium (CAC) score is a well-established predictor of obstructive CAD, proving particularly valuable in identifying high-risk patients independently of clinical risk assessment scores. The utilization of an AI algorithm to compute CAC scores from dedicated non-contrast-enhanced, ECG-gated CT scans has shown to be viable, demonstrating an excellent level of comparability between a fully automated CAC scoring AI application and the traditional semi-automated measurement in a data set of 315 CAC-scoring dedicated CT scans [34]. Several AI methods have demonstrated their capacity to automatically assess the degree of coronary stenosis (CS) directly from image data. The fusion of AI and CAD requires technical expertise, state-of-the-art technologies, and considerable financial and resource investment [34]. AI holds tremendous potential for advancing and enhancing patient care, as demonstrated through ML models that can be used to improve diagnostics accuracy and risk prediction in the realm of non-invasive cardiac imaging and CAD assessment [35].

### 3.2 Applications of AI in Cardiology

AI is rapidly transforming the field of cardiology. In arrhythmias, AI-driven algorithms enhance early detection and diagnostic accuracy by analyzing large electrocardiogram datasets, enabling timely interventions for conditions like atrial fibrillation and ventricular arrhythmias. AI also plays a critical role in valvular heart disease by automating image analysis to assess valve function, predict disease progression, and support clinical decision-making regarding interventions. In echocardiography, AI improves the precision of image acquisition and interpretation, automating the measurement of key parameters like left ventricular ejection fraction and aiding in 3D reconstructions for more accurate diagnoses of complex cardiac conditions. Table **1** demonstrates the use of various AI algorithms in the treatment of cardiovascular diseases.

AI is used to develop innovative tools and solutions to improve heart disease diagnosis, treatment, and management, including arrhythmias. AI algorithms can process vast amounts of clinical data, identify patterns, and predict outcomes, allowing for more precise identification of conditions that are often challenging to detect early. Integrating AI into cardiology makes healthcare delivery more efficient, ensuring better patient outcomes and overall healthcare quality. Fig. (**3**) depicts the AI incorporation in cardiology.

The more fundamental ethical issue is that the algorithmic decisions would perpetuate the existing biases and inequalities, which in turn might result in gender, race, ethnicity, or pathology-specific differential distributions [47]. Imperfect dichotomization and poor calibration are some of the well-known problems connected with the practice of ML in health care applications. During the growth of AI, especially for EMRs, such issues have been prevailing. Such applications of EMRs record medical information but maintain sensitive information on sociodemographic data, including social security numbers and health insurance information. This integration of clinical as well as personal data increases the potential risks associated with improper ML practices and underlines the absolute need for accurate model calibration as well as proper ethical data handling to avoid error on the one hand and protect the privacy of the patient on the other [48].

### 3.3 Cardiac Imaging

CI has emerged as one of the most advanced domains in AI research. Echocardiograms are an excellent tool for non-invasive, quantitative, and qualitative evaluation of cardiac function [49]. AI plays a pivotal role in automating and streamlining the analysis of cardiac images, such as segmenting cardiac structures and quantifying myocardial function [50]. AI-powered image analysis software can segment the left ventricle from echocardiography images with remarkable speed and precision. Arterys Cardio DL, a U.S. FDA-approved software application within the Tempus Radiology division, utilizes DL models to generate automated and modifiable ventricle segmentations from conventional cardiac magnetic resonance imaging (MRI) examinations. These segmentations exhibit accuracy comparable to manual segmentations performed by experienced clinicians. Validation confirmed that the trained DL algorithm produces results within an acceptable error range, similar to that achieved by a proficient clinical expert [51]. Furthermore, AI-powered dose modulation systems have the potential to substantially reduce radiation exposure by as much as 70% during cardiac computed tomography (CT) scans [52]. Philips CT Precise Image utilizes DL for low-dose CT reconstruction, achieving dose reduction, reduced noise, improved low-contrast detectability, and fast reconstruction time. Employing a supervised learning approach and a convolutional neural network (CNN), it replicates the conventional image characteristics typically observed in high-dose filtered back-projection (FBP) reconstructions [53].

Computer-aided diagnostic systems are employed to detect coronary artery stenosis in CT coronary angiography images and assess myocardial infarctions in cardiac magnetic resonance (CMR) images [54, 55]. Similarly, AI is also harnessed for the automated segmentation and quantification of cardiac structures in echocardiography images, enabling the assessment of cardiac function and the diagnosis of various cardiovascular conditions [56]. Developing image and audio-based models typically involves four steps: data quality, classification, measurements, and detection [56]. The initial stage involves the meticulous collection of high-quality data. Caption Guidance is the first FDA-approved AI-powered cardiac ultrasound software that assists clinicians in acquiring diagnostic-quality echocardiographic images in real-time. This software guides users in capturing standard views of the adult heart from various angles during 2D transthoracic echocardiography. However, it is crucial to emphasize that cardiologist review and approval of acquired images remain mandatory for patient evaluation [57]. The next step is the view classification and segmentation of cardiovascular structures. Echocardiographic images require many types of recordings because of the complexity of the cardiac structure. The view classification of cardiovascular structures can be helpful in automated scans or the detection of appropriate views. Several studies have reported a good accuracy for the view classification model for 15 view classifications [56]. Kusunose *et al.* have developed a new view classification model based on 5 predefined views and using a convolutional neural network of 17,000 images with an output error rate of 1.9% mislabeled images [58]. Clustering analyses showed that the neural network could sort heterogeneous input images into five pre-determined views [56]. In addition, to determine if the 98.1% accuracy rate was acceptable for creating a feasible prediction model, they tested the prediction model for EF using the learning data with a 1.9% error rate. The accuracy of the prediction model for EF was warranted, even with training data containing 1.9% mislabeled images [58]. Thus, this approach may provide a clinically feasible method of viewing the classification for the analysis of echocardiographic data. Upon inspection of the misclassified images, they found that many were difficult even for experts to judge, suggesting that DL had been used to successfully imitate human reorganization. Once the image quality has been assessed and appropriate views have been determined, the next step is to measure and quantify the morphological structure. In conventional MLL for EF prediction, after pre-processing images, a human data scientist manually configures a point to focus their attention (region of interest: ROI and segmentation) and extracts features of the target region [59]. Then, the extracted features are used to create a classification model while performing dimension reduction. In DL, all feature extraction steps are embedded in the algorithm, allowing end-to-end learning. The detection of abnormality was the last step. One of the most critical evaluations in echocardiography is the detection of regional wall motion abnormalities (RWMAs). The presence of wall motion abnormalities is directly linked to the treatment decisions. However, the evaluation of regional wall motion abnormalities has been traditionally subjective and relies on visual judgment, such as endocardial motion and myocardial thickness [60]. ML models have been developed to identify and quantify RWMAs. Kusunose *et al.* developed an AI model for the automated detection of RWMAs in myocardial infarction, using a DL algorithm including ResNet, DenseNet, Inception-ResNet, Inception, and Xception for a convolutional neural network. For the detection of the presence of RWMAs, the area under the curve (AUC), which is a performance metric for the DL algorithm, was similar to that of an experienced cardiologist/sonographer (0.97 *vs.* 0.95, *p* = 0.61) and significantly higher than the AUC of resident physicians (0.97 *vs.* 0.83, *p* = 0.003). According to the findings, the detection system of RWMAs may be of great value in the clinical setting [56].

Numerous cloud-based, searchable repositories exist to evaluate AI-based image analysis systems. Notable among them is the Stanford Medical ImageNet, which encompasses more than 230,000 chest radiographs from patients with a variety of clinical conditions [17]. ML models have been created to distinguish between the echocardiographic patterns seen in physiological ventricular hypertrophy, typical of athletes and the findings associated with familial hypertrophic cardiomyopathy [5].

### 3.4 AI During Cardiac Catheterization and Stent Placement

The catheterization laboratory environment is currently undergoing a significant transformation [27]. In recent years, we have witnessed remarkable innovations that have greatly enhanced our capabilities in imaging modalities and methodologies for the analysis and visualization of cardiovascular structures [61]. Robotic interventions, originally developed and utilized in the realm of surgery, are now making their way into the catheterization laboratory [62]. AI functions as a physician assistant, technician, or collaborator in this setting. It issues vocal commands to the interventional cardiologist, ensuring precise positioning of the catheter arm by recommending angles based on the patient's height, weight, and data to yield optimal imaging [23]. The real-time guidance and feedback delivered by AI algorithms during coronary procedures, like angioplasty and stent placement, showcase their ability to predict the physiological response to stent placement accurately [63].

### 3.5 AI During Cardiac Surgery

AI assumes an increasingly important role in surgical decision-making, helping synthesize a broad array of information sources, including patient risk factors, anatomical considerations, disease progression, patient preferences, and cost considerations. This, in turn, assists both surgeons and patients in making more informed predictions about the consequences of surgical decisions [64]. In cardiac surgery, a study has demonstrated the capability of an ML algorithm to surpass the predictive accuracy of the European System for Cardiac Operative Risk Evaluation (EuroSCORE) II and a separate logistic regression model. Specifically, the ML algorithm achieved a remarkable concordance index of 0.795 in predicting in-hospital mortality following elective open-heart surgeries [3]. The utilization of AI has the potential to revolutionize clinical practice. ML empowers the identification of non-linear relationships and previously underappreciated variables, which are traditionally considered to have limited utility. This ability to incorporate complex data patterns marks a transformative shift in clinical decision-making. Fig. (**4**) represents the swift image analysis by AI showcasing the rapid and accurate interpretation of medical images, which aids in timely diagnosis and decision support in cardiac surgery. By analyzing large datasets of patient images, the AI system identifies subtle patterns that contribute to more precise and individualized risk assessments, further enhancing surgical outcomes.

### 3.6 AI Diagnostic Solution to Assess Future Risk of Cardiovascular Disease

Taylor *et al.* [65], employed DL to develop a cardiovascular risk stratification system predicting coronary artery calcium (CAC) using retinal photographs. CAC score, a validated marker of cardiovascular disease risk, was comparable between DL-derived retinal CAC (RetiCAC) and CT scan-measured CAC in predicting cardiovascular events. This suggests retinal photograph-based DL as a potential alternative for CAC assessment, particularly in resource-limited settings.

The DL algorithm was trained on 216,152 retinal images from various datasets (South Korea, Singapore, UK) to predict CAC presence probability (RetiCAC score). Stratified into tertiles, RetiCAC was evaluated using Cox proportional hazards models for predicting CVD events in external datasets from the aforementioned regions and the UK Biobank. Additionally, the incremental value of RetiCAC, when combined with the pooled cohort equation (PCE), was assessed in the UK Biobank participants [65].

A two-stage DL architecture was implemented for retinal image analysis. Firstly, a pre-trained EfficientNet model served as a convolutional feature encoder, extracting relevant features from retinal images after preprocessing (cropping and resizing). This resulted in separate feature maps for each eye. Global average pooling then discarded spatial information, generating 160-dimensional feature vectors. Subsequently, these feature vectors from both eyes were concatenated, forming a 320-dimensional representation. Finally, a fully connected layer with sigmoid activation produced a single probability score, indicating the likelihood of the input belonging to the abnormal class (having a non-zero CAC score). This approach effectively leverages pre-trained features for efficient and robust retinal image analysis. A sigmoid function transforms the model's output to a probability between 0 and 1 [66].

The convolutional feature encoder was augmented with additional fully connected layers to capture inter-ocular relationships. Batch normalization enhanced training stability and speed. Preprocessing included contrast enhancement, and data augmentation employed random cropping, flipping, rotation, and brightness/saturation adjustments to prevent overfitting. An Adam optimizer with fixed weight decay, a learning rate of 0.0001, and a batch size of 32 were used for training over 200 epochs. The resulting model predicts the probability of CAC presence, a key biomarker [65].

### 3.7 Applications of AI in Arrhythmias

AI has the potential to revolutionize the diagnosis and management of arrhythmias, with the following key applications:

#### 3.7.1 Arrhythmia Detection

Cardiac arrhythmias impact approximately 2% of adults residing in communities, with an annual incidence rate of about 0.5%. These arrhythmic manifestations encompass relatively benign conditions, such as atrial and ventricular premature contractions, as well as serious and life-threatening arrhythmic conditions like ventricular tachycardia (VT) and ventricular fibrillation (VF), which can lead to sudden cardiac death (SCD) [67]. Arrhythmias can be identified through an ECG by examining deviations in the heart's rhythm and rate [68]. Several novel feature extraction methods, incorporating higher-order statistics, wavelets, morphological descriptors, and R-R intervals, were employed to decompose ECG signals. These features were fed as a sequence to a single long short-term memory network (LSTM) model, achieving high performance in classifying five arrhythmic rhythms and normal beats. The model attained precision, accuracy, specificity, F-score, and sensitivity of 96.73%, 99.37%, 99.14%, 95.77%, and 94.89%, respectively [68]. Early detection of arrhythmias is crucial for averting these complications. ECG measures the electrical activity of the heart and can be conducted while the patient is at rest or during physical activity [69]. The substantial amount of ECG data collected daily, in-home and hospital settings, may pose challenges for human operators/technicians when it comes to data review [70].

#### 3.7.2 ECG Analysis

##### 3.7.2.1 Automated Detection of Arrhythmias

AI tools have demonstrated their potential in automating and aiding disease diagnosis, and there is ongoing development of tools designed to enhance disease prognosis prediction and therapeutic response as well as provide new insights into health and disease characterization. AI algorithms are trained to identify patterns within ECG signals that signify specific arrhythmias [71]. This advancement holds the potential to enhance the accuracy and efficiency of arrhythmia detection, making it more accessible to patients [72]. ML algorithms are utilized to train models on extensive datasets of ECG recordings, encompassing both normal and arrhythmic cases to enable the identification of arrhythmias in new ECG recordings [70]. The DL algorithms are a type of ML algorithm that excels at learning complex data patterns and have proven highly effective in arrhythmia detection, with an increasing presence in clinical practice [73]. Cardiologist, a cloud-based, vendor-neutral AI-powered arrhythmia diagnostic software, utilizes a clinically validated algorithm to analyze ECGs. It detects over 20 cardiac events and parameters, including PVC morphology, QTc, and heart rate variability (HRV), aiding in comprehensive clinical interpretation. Trained on a vast ECG database, its DL architecture reliably captures ventricular and atrial rhythms, including P wave analysis, for enhanced specificity compared to traditional programs (171). Furthermore, AI-enabled ECG algorithms are effectively employed in forecasting the recurrence of paroxysmal AF following catheter ablation [74].

#### 3.7.3 Classification of Different Types of Arrhythmias

The remarkable success of ML algorithms in classifying cardiac arrhythmias is truly impressive. The efficacy of classification results hinges on various factors, including heart rate variability, the distinct morphology of the ECG curve (including low voltage flutter waves corresponding to atrial depolarization), or a combination of both. This fusion of features allows for a more comprehensive understanding of the underlying arrhythmias, leading to more accurate predictions and better clinical outcomes. Thus, the deployment of ML in cardiology, especially in arrhythmia detection, continues to revolutionize the field, offering faster, more reliable diagnoses that were previously unattainable with traditional methods.

Sager *et al.* propose a three-phase, biology-informed ML approach for distinguishing atrial fibrillation (AF) from atrial flutter (AFL) using surface ECGs. This method leverages deep domain knowledge to generate clinically interpretable features through mathematical optimization. These features, derived from the same ECG data, capture the pathophysiology of atrial flutter and are subsequently integrated into an ML model. This approach achieves exceptional accuracy (82.84%) and ROC AUC (0.9), surpassing previous methods. Notably, the domain knowledge component utilizes a tailored branch-and-bound algorithm, while standard algorithms like Adam (Adaptive Moment Estimation) can be employed for training the ML model [75].

Cardiac arrhythmias are commonly categorized as bradyarrhythmia and tachyarrhythmia. Both types have the potential to reduce cardiac output, leading to hypotension and ultimately, they can be fatal in certain circumstances, yet they involve different underlying mechanisms [76]. Bradyarrhythmias are characterized by abnormally slow heart rates [77]. The most prevalent form of bradyarrhythmia is sinus bradycardia, typically arising from a decreased automaticity of the sinoatrial node, the heart’s natural pacemaker [78]. Atrioventricular (AV) block is another type of bradycardia resulting from a delay or blockage in the conduction of electrical signals from the atria to the ventricles. Complete blockage in this conduction constitutes a condition called complete heart block [79]. Tachyarrhythmias, on the other hand, are abnormal heart rhythms that are characterized by a rapid heart rate. Atrial fibrillation, a specific type of tachyarrhythmia, is marked by rapid, irregular electrical signals in the atria [80]. Atrial flutter is another type of tachyarrhythmia. Supraventricular tachycardia, ventricular fibrillation, and ventricular tachycardia represent different types of tachyarrhythmia. They arise from abnormal electrical signals in the atria or AV node, rapid, chaotic electrical signals in the ventricles, and rapid regular electrical signals in the ventricles, respectively [81].

### 3.8 Different AI Strategies

These AI-driven systems, supported by significant advances in computational power, are enhancing the precision, speed, and efficiency of diagnostic and therapeutic practices across various medical specialties. The integration of AI technologies into clinical workflows is poised to revolutionize patient care by facilitating more accurate decision-making, optimizing treatments, and reducing the burden on healthcare professionals [82]. As a more significant sub-field of AI, ML, or ML, enables algorithms to learn and interpret data automatically. The recent innovations of AI, especially in ML, dramatically influence biomedical science by bringing forth new predictive techniques and modeling that can be applied to various clinical conditions [83]. AI-driven image and signal analysis has achieved human-level performance in various applications, primarily due to advancements in modern ML, particularly deep learning with convolutional neural networks. In comparison to other technological fields, deep learning in healthcare is still emerging, with its application to cardiology remaining relatively limited. The initial commercial uses of deep learning were centered around computer vision, particularly for image analysis [1]. Conversely, in deep learning frameworks, the representation of the input data is acquired autonomously by the network. The predominant architecture employed for this form of representation learning is a specific subclass of neural networks known as Convolutional Neural Networks (CNNs) [31].

### 3.9 Ambulatory Monitoring Devices

#### 3.9.1 Wearable Devices in Cardiac Monitoring

Ambulatory monitoring devices are gaining popularity for continuous cardiac disease monitoring. Recent advancements have resulted in cost-effective and reliable solutions that enable the continuous monitoring of vulnerable populations from the comfort of their homes [84]. Wearable devices are defined as those worn on the human body or clothing. They typically consist of a target receptor and a transducer. The receptor recognizes a specific analyte and responds accordingly, while the transducer converts the receptor’s response into a useful signal. Wang *et al.* conducted a study proposing an energy-efficient wearable intelligent ECG monitor scheme with a two-stage end-to-end neural network and diagnosis-based adaptive compression. Compared to the state-of-the-art solutions, this scheme significantly reduces the power consumption in ECG diagnosis and transmission while maintaining high accuracy [85]. The ECG WATCH, developed in the Neuronica Lab of the Politecnico di Torino, is a wearable, wireless, non-invasive device designed to easily record 10-second single-lead ECGs and visualize them on a smartphone or desktop app. The recorded data can be sent to a physician for analysis to determine if further examination is needed [86].

In another study, Lee *et al.* presented a highly flexible epidermal design for a novel ECG and heart rate logging wearable sensor called “WiSP”, this is a low-cost, light-weight (1.2 g) device comparable in size to a standard adhesive bandage (58 mm × 25 mm × 1 mm), streams physiological data to commercial smartphones via standard near-field-communication (NFC) for use in both ambulatory and home-based settings [87]. Steinberg *et al.*, introduced a novel form of wearable ECG sensors that provide an alternative tool for long-term rhythm monitoring, potentially offering increased sensitivity to detect intermittent or subclinical arrhythmias [88].

#### 3.9.2 AI Algorithm for Real-time Arrhythmia Detection

AI-based methods have demonstrated remarkable success in various aspects of ECG signal processing, including denoising, quality assessment, characteristic wave detection, delineation, and arrhythmia classification. DL approaches leverage deep representation features and temporal information from ECG signals to classify heartbeats or rhythms [89]. In a study by Marsili *et al.*, the feasibility of real-time AF detection using a wearable ECG device was demonstrated. The onboard implementation of this wearable device achieved exceptional real-time performance. Boasting an accuracy of 98% with minimal memory usage (less than 6 kB) and swift computation time (less than 0.2 mis per beat) [90]. Farag MM proposed another novel method for ECG classification, optimized for edge deployment and suitable for embedding in a wearable device for arrhythmia detection. This method offers a clear interpretation supported by visualizations of the Conv1D layer operation as a finite impulse response (FIR) filter and utilizes this insight to create a self-contained short-time Fourier transform (STFT) ECG classifier. The Conv1D layer is a component in a neural network designed to analyze one-dimensional sequential data, such as ECG. The FIR filter is a digital filter that processes input signals through a convolution operation and has a finite duration of response to an input impulse. It is readily deployable for real-time ECG monitoring and arrhythmia detection, even on resource-constrained edge devices [91]. Eko Health offers a commercially available digital stethoscope equipped with AI-powered arrhythmia detection. A clinical study demonstrated the Eko AI's ability to identify AF with high sensitivity (99%) and specificity (97%) when analyzing the single-lead ECG tracing integrated within the Eko DUO stethoscope. This integrated ECG capability empowers healthcare providers to efficiently screen patients for clinically relevant arrhythmias during routine physical examinations (176).

### 3.10 Risk Stratification and Prognosis

#### 3.10.1 Identification of High-risk Patients

The identification of high-risk patients for arrhythmias is a critical aspect of cardiovascular care, as arrhythmias can lead to severe complications [92]. Clinically, a thorough examination of the patient's medical history is essential, including a focus on family history, previous episodes of arrhythmias, and the presence of underlying heart conditions [93]. Within the complex terrain of chronic heart failure (CHF), it becomes increasingly evident that our current prognostic tools often fall short of comprehensively considering the nuanced interplay of sex-specific factors [94]. AI algorithms process vast amounts of clinical and imaging data, providing more nuanced insights into patient-specific factors and risk stratification [95]. The intersection of chronic obstructive pulmonary disease (COPD) and acute myocardial infarction (AMI) unravels a complex paradigm of risk stratification [96]. Echocardiography and cardiac MRI emerge as potent tools, especially in identifying high-risk patients post-AMI [97]. ECG and cardiac MRI emerge as powerful tools, particularly in identifying high-risk patients post-AMI predisposed to arrhythmic events [98]. In the evaluation of infarct size and resting LV function, transthoracic echocardiography takes center stage as the most suitable and readily accessible technique. AI assists in the automated analysis of images, helping clinicians to quickly and accurately evaluate infarct size, LV function, and right ventricular (RV) involvement. These imaging modalities contribute valuable insights into the structural and functional aspects of the heart, aiding in the identification of patients at risk for arrhythmias [99]. Meanwhile, cardiac MRI, though considered the gold standard, grapples with accessibility constraints [100]. In the complicated ballet of post-AMI risk assessment, RV systolic function emerges as a critical player. RV involvement, often lurking in the wake of an inferior AMI, carries profound prognostic implications [101]. Various echocardiographic parameters, such as TAPSE, fractional area change, and RV strain, are meticulously employed to gauge RV function and to prognosticate with precision [102]. This approach to risk assessment, encompassing both LV and RV function, provides an understanding of the cardiovascular landscape, offering valuable insights into the potential arrhythmic vulnerabilities of patients post-AMI within the broader context of chronic heart failure [99]. AI tools potentially address the accessibility constraints associated with cardiac MRI [103]. In fact, a study explored the potential of DL models trained on routine 12-lead ECGs to predict AF within 31 days across diverse populations. Hospitals using the trained convolutional neural network achieved high accuracy in various patient groups, demonstrating its effectiveness across demographics and comorbidities. This approach holds promise for future AF screening to mitigate associated complications [104]. By automating the analysis of imaging data, AI expedites the interpretation process, allowing for quicker and more widespread utilization of advanced imaging techniques, even in settings where accessibility might be a challenge [105]. A study conducted by Akshay *et al.*, using a novel vision-text transformer for automated complete diagnosis generation from ECG, highlighted that AI-ECG can diagnose a multitude of rhythm and conduction disorders. For the study, they developed an ECD-specific vision encoder-decoder model with a pretrained BEiT transformer encoder with a 384 x 384-pixel input size and a generative pretrained transformer, GPT 2, as a decoder. The model performed well across conventional Natural Language Processing performance metrics, with ROUGE-L and BLEU-1 scores of 0.695 and 0.502, respectively, and for clinical diagnosis detection performance metrics (>90%) for various conditions, including AF [106].

#### 3.10.2 Predictive Models for Assessing the Likelihood of Arrhythmia Occurrence

AI is currently at the forefront of transformative efforts in the field of AF, a widespread arrhythmia associated with substantial morbidity. The integration of patient clinical characteristics into multivariable prediction models, exemplified by the CHARGE-AF score, undergoes sophisticated refinement *via* AI and ML methodologies, leveraging electronic health record (EHR) datasets. The emergence of AI-Enhanced ECG (AI-ECG) as a valuable tool for AF prediction is evident, showcasing initial algorithms that exhibit noteworthy accuracy in detecting paroxysmal AF. The exploration of AI/ML techniques for risk stratification and prognosis prediction in AF patients encompasses alternative methodologies, including intracardiac signals, chest radiography, and facial PPG signals [107, 108]. Ongoing research endeavors are dedicated to elucidating the appropriate clinical implementation of AI/ML-enhanced tools while systematically addressing concerns related to diversity, economic barriers, and perceptions of both patients and physicians. The promising application of AI in AF diagnosis and treatment necessitates judicious consideration to ensure equitable and transparent implementation. The intrinsic suitability of AI for AF risk estimation, given its adept utilization of common diagnostics such as ECGs, serves as a cornerstone in informing preventive interventions [108, 109]. In the broader landscape of cardiovascular healthcare, AI assumes a pivotal role in predicting and preventing ventricular arrhythmias and sudden cardiac arrest (SCA), representing a paramount global public health concern. Despite advancements in acute management, SCA remains predominantly lethal, with at least 90% of cases culminating in sudden cardiac death (SCD). Multimodal AI tools, which combine data from different sources, present a promising method for risks assessment and stratification. Specific cardiac conditions such as hypertrophic cardiomyopathy (HCM), dilated cardiomyopathy (DCM), long QT syndrome (LQTS), and Brugada syndrome (BrS) attract focused attention, with ML models adept at predicting the risk of sudden death in these populations [110, 111]. The purview of AI extends to left ventricular hypertrophy (LVH), where algorithms showcase commendable accuracy, precision, sensitivity, and specificity, particularly in the identification of LVH. The application of AI encompasses the detection of left ventricular systolic dysfunction, utilizing both traditional 12-lead ECGs and single-lead ECGs derived from smartwatches. The generation of sensitivity maps by AI plays a pivotal role in the identification of critical features, contributing to the precision of classification. AI algorithms centered on ECG data manifest efficacy in risk prediction for diverse cardiovascular events, enhancing accuracy through the seamless integration of ECG features with pertinent clinical variables. Notably, AI models leveraging ECG data contribute significantly to the classification of patients into high- and low-risk subgroups, optimizing patient selection for device therapy [111, 112].

Survival Study of Cardiac Arrhythmia Risk (SSCAR) is a novel DL framework for predicting patient-specific risks of SCD. It integrates multiple custom neural networks that fuse diverse data types, such as cardiac magnetic resonance (CMR) images and clinical covariates. Statistical survival analysis enables the estimation of individualized survival curves with accurate SCD probabilities over a 10-year horizon. The statistical framework ensures reliable integration of these features for accurate survival prediction [113].

### 3.11 AI-guided Intervention and Therapy

#### 3.11.1 Catheter Ablation (CA) Procedures

CA is a minimally invasive medical procedure used to treat certain heart rhythm disorders, particularly supraventricular tachycardia. This procedure involves the use of a catheter, to correct or disrupt abnormal electrical pathways in the heart, restoring normal heart rhythm [114]. It is a crucial strategy for managing AF and reducing its associated complications [115]. The recurrence rates following CA vary based on the type of AF, with paroxysmal AF recurring in 10-30% of cases within 12 months, and persistent AF recurring in 25-35% of cases [116-118]. A study revealed that recurrence occurred in 13.5% of patients, underscoring the need for effective methods to identify and manage the risk of recurrence after CA [119]. The risk of recurrence after CA is influenced by multiple factors, such as the duration of AF, patient age, left atrial (LA) size, renal insufficiency, atrial fibrosis, and the expertise of the operators involved [120]. The emergence of DL methodologies has facilitated the integration of AI algorithms into the domain of CVD [121]. A recent study introduced an AI-enabled ECG algorithm capable of predicting the risk of recurrence in patients with paroxysmal AF after CA. This innovative AI algorithm not only aids in the diagnosis of ECGs but also demonstrates its effectiveness in predicting the prognosis of AF using a 12-lead ECG. This predictive tool has the potential to identify patients who may benefit from catheter ablation and guide the selection of appropriate ablation strategies [121]. Catheter ablation, primarily through pulmonary vein isolation (PVI), is an effective means of restoring sinus rhythm in patients with AF. However, the prognosis following this therapy is intricate and encompasses aspects like recurrence or recurrence-free outcomes, as well as short-term and long-term recurrence [122]. Additionally, previous work has applied ML to predict AF ablation recurrence using shape descriptors extracted from magnetic resonance imaging and by combining imaging and clinical biomarkers. Furthermore, ML methods, in conjunction with personalized computational modeling, have shown promise in predicting PVI recurrence [123]. This improved narrative integrates detailed aspects of recurrence risks, the role of AI in prediction, and its clinical significance. Fig. (**5**) shows the catheter ablation procedure with the significance of AI.

#### 3.11.2 Pre-Procedure Planning using AI Algorithms

Pre-procedure planning using AI algorithms is an emerging field within the broader context of AI-guided intervention and therapy [124]. AI serves as a valuable tool for aiding healthcare practitioners in planning medical procedures, such as surgeries or ablation procedures [125]. AI algorithms are adept at scrutinizing patient data, which frequently encompasses medical imagery such as CT scans or MRIs, to facilitate the formulation of precise treatment plans [126]. This involves segmenting relevant structures or tissues, identifying the best approach, and estimating the optimal parameters for the procedure. Moreover, AI can also lend its capabilities to predict potential complications or clinical outcomes based on historical data, thereby enhancing the decision-making capacity of medical professionals [127]. AI algorithms analyze electroanatomic maps and identify the areas responsible for initiating or perpetuating the arrhythmia, assisting in planning ablation strategies to target specific regions effectively [128]. In the case of device implants, such as pacemakers or defibrillators, AI predicts the likelihood of future arrhythmias based on historical data. This information guides clinicians in determining the appropriate device settings and programming to prevent or respond to potential arrhythmic events [129]. AI algorithms analyze electrophysiological data to stratify patients based on the risk of arrhythmia recurrence or complications, which helps ensure a more targeted and effective approach for specific interventions [130]. AI can assist in optimizing cardiac resynchronization therapy (CRT) device programming to enhance ventricular synchrony and improve overall cardiac function in heart failure patients with arrhythmias [131].

#### 3.11.3 Real-time Guidance During Ablation Procedures

Real-time guidance during ablation procedures is another application of AI in healthcare. In the context of cardiac ablation procedures, AI is an invaluable tool, offering immediate feedback to the performing physician [132, 133]. This feedback stems from a variety of data sources, including electroanatomic maps, 3D imaging, and other sensory inputs [134]. AI models, such as Mask2Former, automate the measurement of ablation zones from ultrasound images, significantly reducing variability associated with manual measurements [135]. AI algorithms play a pivotal role in directing the ablation catheter precisely toward the intended target area, thereby ensuring precise and effective therapy [136]. Furthermore, the technology can continuously monitor the ongoing procedure and provide alerts or recommendations based on the data being collected during the operation. Real-time guidance significantly enhances the precision and safety of ablation procedures [137]. In the context of atrial fibrillation treatment, catheter ablation involves the use of a catheter to deliver radiofrequency or cryotherapy to specific areas of the left atrium to create scars or lesions [138]. The Volta VX1 software utilizes ML to assess spatiotemporal dispersion in atrial fibrillation patients, leading to better-targeted ablation strategies compared to traditional methods [139, 140]. Real-time guidance positions the catheter at the entrance of the pulmonary veins, where the abnormal signals typically originate [141]. Ablation procedures are performed in ventricular tachycardia to target the areas causing the abnormal rhythms and the catheters are navigated to the specific locations within the ventricles where the abnormal electrical circuits are located [142]. The ablation procedures that are performed for atrioventricular nodal reentrant tachycardia (AVNRT) involve disrupting abnormal electrical pathways in the atrioventricular node and real-time guidance identifies and ablates the specific pathways responsible for the reentrant tachycardia [128]. The Rhythmia HDx Mapping System integrates cutting-edge hardware, a proprietary high-throughput algorithm, and the INTELLAMAP ORION™ mapping Catheter to deliver unparalleled high-definition cardiac mapping capabilities, enabling the management of even the most intricate electrophysiological procedures [143].

### 3.12 Implantable Devices

#### 3.12.1 Advances in Cardiac Implantable Electronic Devices and Remote Monitoring

In recent years, there has been a remarkable uptick in the utilization of advanced cardiac implantable electronic devices (CIEDs), including pacemakers, implantable cardioverter-defibrillators (ICDs), and cardiac resynchronization therapy (CRT) devices, fundamentally reshaping the field of cardiac healthcare [144]. The prevalence of remote monitoring (RM) for CIEDs, encompassing permanent pacemakers (PPM), ICDs, and CRTs, is on an impressive upward trajectory [145]. In particular, RM stands as a promising avenue for patients facing travel challenges or residing at a considerable distance from healthcare facilities. It facilitates the collection and transmission of vital device data to medical professionals via phone or internet connectivity, offering unprecedented advantages in patient care [146]. Normally, patients with devices like ICDs, CRTs, or pacemakers visit clinics for check-ups on a regular basis. Remote monitoring simplifies this by securely transferring device data online, and using an alert system can help manage the increased demands of remote monitoring [145, 147]. Patients most vulnerable to sudden cardiac arrest often bear the burden of coronary heart disease and reduced left ventricular EF. The ICD emerges as a pivotal intervention and acts as a remarkable device that diligently monitors the intricate rhythms of the heart, swiftly intervening with anti-tachycardia pacing and cardioversion or defibrillation when deemed necessary [148]. Heart failure carries significant health implications, accompanied by a substantial burden of morbidity and mortality, often necessitating hospitalization, and plays a formidable role in the realm of sudden cardiac death. The use of implantable cardiac resynchronization with defibrillator capabilities (CRT-D), which combines pacing therapy with a defibrillator, is considered a highly effective strategy for managing complex cardiac arrhythmias and heart failure [149]. In the realm of cardiovascular therapeutics, the deployment of a permanent pacemaker takes center stage as the implant remedy for cases marked by symptomatic bradycardia. The use of a permanent pacemaker addresses an array of conditions, encompassing sinus node dysfunction and atrioventricular block, and extending its vigilant embrace to high-risk asymptomatic individuals with cases of bradycardia-related complications [150]. RM, spanning its boundaries from diligently timed data transmissions, stands as an indispensable cornerstone within contemporary CIED management paradigms [151].

#### 3.12.2 Proactive Monitoring for Cardiac Implantable Electronic Devices Safety

Early detection of device malfunction or complications is a critical aspect of managing patients with CIEDs. The early detection of device-related issues is important and can prevent serious health risks and improve patient outcomes. By identifying the problem, we can prevent situations that could compromise the quality of care these devices provide to patients [152]. Lead malfunctions, resulting from lead fractures or insulation breaks, are common complications. This can lead to ineffective pacing or defibrillation. All cardiac electronic devices are powered by batteries that have a limited lifespan. Early detection of battery depletion allows for proactive device replacement before the battery reaches the end of service. Infections can occur at the implant site or along the device leads. Devices can sometimes become dislodged from their intended position. Early detection helps in repositioning or replacing the device as needed [152, 153]. Remote monitoring allows continuous surveillance of the device's performance. It includes routine transmissions and alerts triggered by device malfunctions, arrhythmias, or other anomalies [154, 155]. Timely intervention can lead to better patient outcomes and a reduced risk of complications, including infections and device-related adverse events. Patients with well-functioning devices experience fewer disruptions to their daily lives, reduced anxiety, and improved overall quality of life [145, 156]. Within the intricate tapestry of cardiac electronic device care, the crucial role of early detection stands as an indomitable part. It is a symphony associated with the harmonious integration of remote monitoring, in-person evaluations, patient education, and swift clinical interventions that work in unison to safeguard the well-being of individuals entrusting their lives to these technological advancements [157].

## 4 DISCUSSION

### 4.1 Benefits of AI in Cardiology

#### 4.1.1 Improved Accuracy and Efficiency of Diagnosis

The potential of ML, DL, and CC to change the way cardiac data is utilized -their accuracy, objectivity, and efficiency - is undeniable. The efficacy of ML in the analysis of predefined tasks, such as image processing for border detection on a scan or recognizing suspicious areas on an image, is a crucial aspect of ML’s utility in healthcare [158]. The use of AI in cardiovascular medicine improves patient care, increases efficiency, and enhances clinical outcomes [159]. The development of a multilabel automated diagnosis model for electrocardiographic images overcomes the limitation of reliance on the infrequently available signal-based data [160]. In the field of imaging, the progress of AI has been enormous in the last few years, affecting all the phases of the diagnostic process. The use of enormous sets of digital ECGs connected to detailed clinical data to create AI algorithms for the detection of silent (previously asymptomatic and undocumented) AF, LV dysfunction, and hypertrophic cardiomyopathy, in addition to the ability to determine a person’s age, race, and sex, amongst other phenotypes [159]. Hannun *et al.* in a study demonstrated the efficacy of DL in the classification of various ECG rhythms, a powerful addition to every cardiologist’s diagnostic toolkit [161]. Gadaleta *et al.* show that a deep learning approach integrating morphologic analysis of single-lead ECG without AF has significantly improved near-term AF prediction. This advancement could support a digital strategy to assess individuals and provide a risk score for future AF, allowing for more targeted extended monitoring of those at higher risk [162]. The use of AI in this regard may help standardize the quality of care by helping to reduce two major elements of decision-making error: social bias and noise, factors that may affect a physician’s judgment [158]. AMI risk classification has been greatly aided by ML, with popular algorithms such as logistic regression (LR), support vector machines (SVM), K-nearest neighbor (KNN), artificial neural network (ANN), and random forest (RF). ML falls into three distinct types: supervised (labeled data), unsupervised (unlabeled data), and reinforcement (feedback from rewarding behaviours). These advanced techniques help quickly analyze large amounts of medical data and reveal hidden structures, allowing for a more efficient and semi-automated approach to finding patterns in data [163]. AI is ready for the prime-time field of cardiology. Prior to the advent of information technology, researchers primarily relied on traditional statistical methods to infer conclusions from their findings. Linear regression was a cornerstone of these approaches, later evolving into logistic regression, ridge regression, and other techniques tailored to specific research questions. Common risk prediction models for CVD included Cox Proportional Hazard, Framingham Risk Score, TIMI, SCORE, GRACE, QRISK, and HEART. While these models have varying focuses, many share the goal of predicting 10-year CVD risk. Recent advancements in QRISK, incorporating cerebrovascular and immune disease parameters, aim to enhance its predictive accuracy. Traditional methods often rely on a “one-size-fits-all” approach. AI, particularly ML, and deep learning, offers a promising avenue for developing personalized predictive models that can provide tailored guidelines for cardiologists and improve patient-centered decision-making. Ultimately, these advancements can streamline clinical workflows and enhance the quality of patient care [164].

AI-driven classification addresses the diverse classification that encompasses the AF detection and subtype stratification and predicts the incident and recurrent AF [165]. AI-driven techniques streamline diagnosis by automating complex data analysis, ultimately improving patient outcomes through quicker and more accurate medical insights. Transfer learning, a form of CNN, accelerates AI training by leveraging pre-trained models, thus enhancing performance in tasks like early disease detection with limited data. Moreover, advanced ML algorithms like random forest (RF), extra trees (ET), and stochastic gradient boosting (SGB) excel in classifying vast datasets, providing robust, interpretable models for clinical decision support. Algorithms such as artificial neural networks (ANN), especially multilayer perceptron (MLP), study intricate relationships in patient data to improve diagnosis predictions. Ensemble methods, including Ada Boost Classification (ABC) and boosted ensemble algorithms, combine multiple classifiers to enhance accuracy, offering clinicians more reliable diagnostic tools. Table **2** demonstrates how AI is beneficial in improving the accuracy and efficiency of diagnosis [166-174].

#### 4.1.2 Enhance Risk Stratification and Patient Management

AI has significantly improved risk stratification and patient management in cardiology by leveraging ML algorithms and large datasets to provide more accurate, personalized, and timely care [175]. AI algorithms play a pivotal role in predicting cardiac events, facilitating early intervention, and targeted treatment for individuals at risk [176]. AI continuously monitors and analyzes patient data, leading to the identification of high-risk individuals [177]. AI-driven approaches offer the potential to enhance patient care and improve clinical outcomes. Genetic profiling is a fundamental component where AI algorithms analyze genetic data to identify genetic markers associated with drug response and disease susceptibility. This genetic information serves as a guide for tailoring medications and interventions to enhance their effectiveness for specific patients [178]. In cases where a patient with heart disease experiences adverse effects from a particular medication, AI can recommend alternative options, ensuring the most suitable medications and dosages [179]. Moreover, AI-powered personalized medicine has the potential to optimize healthcare costs. By eliminating the inefficient trial-and-error process often associated with traditional treatment methods, AI allows healthcare professionals to rapidly identify the most effective treatment options for each patient. This reduces the need for unnecessary tests and hospitalizations, ultimately benefitting both patients and healthcare institutions [180, 181]. AI extends its capabilities to lifestyle recommendations, integrating factors such as diet and exercise habits into personalized treatment plans [182]. AI systems exhibit the capability to detect early-stage abnormalities such as structural heart defects or coronary artery blockages, enabling timely interventions [126]. AI also plays a pivotal role in blood biomarker analysis, assessing blood biomarkers associated with heart disease. AI-driven algorithms can discern patterns in blood tests, including elevated cardiac troponin levels, which signify conditions like MI, ultimately facilitating early detection and intervention [183].

#### 4.1.3 Increased Accessibility and Affordability of Care

AI technologies substantially impact the accessibility and affordability of cardiology care by expanding the reach of services and streamlining healthcare operations [184]. AI offers potential solutions by streamlining image acquisition, interpretation, and decision-making, thereby reducing costs and enhancing value. While ML development costs have been overlooked, initial advancements have required substantial software, IT infrastructure, and clinical data. Continued progress towards more complex ML models will necessitate significant investment [185]. AI-driven telemedicine and remote monitoring solutions have transformed the landscape of cardiac care, enhancing accessibility and convenience for patients. AI-powered systems support real-time data transmission, enabling cardiologists to remotely assess patient conditions and provide recommendations [186]. AI algorithms diligently analyze data collected from remote monitoring devices, smartwatches, and continuous ECG monitors, engage in self-monitoring of their cardiac health, and effectively identify irregularities [134, 187]. AI-automation significantly diminishes the need for labor-intensive manual interpretation, subsequently reducing diagnostic costs and expediting results [134]. AI algorithms contribute to enhanced efficiency by collaborating with radiologists and cardiologists to highlight specific areas of concern in medical images, elevate diagnostic accuracy, and minimize the time required for image interpretation, leading to substantial cost savings [188]. AI models enable healthcare systems to allocate resources optimally and curtail unnecessary diagnostic costs, ultimately streamlining the diagnostic process [14]. Predictive analytics, driven by AI algorithms, enable hospitals to forecast patient admission rates and resource utilization [189]. AI-powered appointment scheduling systems contribute to this optimization by minimizing wait times for cardiology appointments. These systems achieve this by adeptly allocating appointment slots based on patient needs and physician availability [190]. AI's role in resource allocation presents significant potential to enhance the healthcare system's effectiveness and cost-efficiency. AI identifies individuals at risk and recommends early interventions and preventive measures. It achieves this by analyzing electronic health records to pinpoint patients with risk factors for heart disease and subsequently prompting physicians to provide counseling and interventions [177]. Remote monitoring facilitates personalized guidance and recommendations, empowering patients to proactively manage their cardiac health [182]. The integration of AI in these aspects of healthcare underscores its potential to alleviate the burden on the healthcare system by promoting early interventions and preventive measures.

### 4.2 Limitations of AI in Cardiology

#### 4.2.1 Data Quality and Availability

AI has revolutionized the field of cardiology in the context of data quality and availability with its widespread reach. The trained models of AI correlate with the quality of data that is incorporated for processing, and in turn, they provide the outcomes of the data being processed. The perpetuation of the existing biases in the healthcare system must be taken into consideration in the case of AI models. Training issues also limit the success of ML. If the images are poorly lit, ECGs and cardiac CT scans can lead to inaccurate predictions. ECG has its limitations, such as the inability to provide spatial references for evaluating structural changes in the atria or visual guidance for invasive procedures. AI models need to be trained on a large and complete dataset to learn the complex relationships between different variables. The algorithms developed by ML technologies are only as effective as the data used to frame them [191]. The limitations of AI models include the differences among the validation cohorts and the possibility of overestimating the risk of CVD. AI language is still in its early stages of application to cardiology aspects. Relying solely on a language model’s recommendation could lead to misdiagnosis or incorrect treatment decisions, potentially harming patients.

#### 4.2.2 Regulatory and Ethical Considerations

AI is a rapidly evolving field, and regulators are struggling to keep up with the pace of innovation. As a result, there is a risk that AI systems could be developed and deployed without adequate safety and efficacy testing. AI systems are trained on data, and if the data is biased, the AI system will learn these biases and produce biased results [192]. This could lead to disparities in the quality of care that patients receive. There are also several ethical considerations surrounding the use of AI in cardiology. For example, it is important to ensure that patients have informed consent before their data is used to train AI models. It is also important to ensure that AI systems are used in a way that respects patient autonomy and privacy. Regulatory bodies often struggle to keep pace with rapidly evolving AI technologies, which lead to the timely approval and integration of AI solutions in cardiology and healthcare. Regulatory bodies, like the American Food and Drug Administration, have difficulty with the regulation and approval of software based on AI. Regulatory frameworks can differ between countries, leading to fragmentation in the implementation of AI applications and creating barriers to global healthcare collaborations and interoperability. AI systems can be vulnerable to data breaches, potentially exposing sensitive medical information. Ensuring robust cybersecurity measures is a continuous challenge. Ensuring AI algorithms' clinical validity and safety is complex, resource-intensive, and often lags behind AI development. The AI systems can perform well in controlled settings but may not replicate the same performance in real-world clinical scenarios, which leads to ethical concerns and necessitates a comprehensive evaluation. The determination of legal responsibility in cases of AI-related medical errors is an ethical and regulatory challenge. The proliferation of AI tools and applications in healthcare has resulted in some falling through regulatory gaps, posing risks to patient safety and ethics.

#### 4.2.3 Integration with Existing Clinical Work Flows

Existing clinical systems, such as EHRs and medical devices, often use different standards and data formats. AI applications must be compatible with these systems and achieving interoperability can be complex and time-consuming. AI models require access to large and diverse datasets for training and validation. Integrating AI into clinical workflows requires financial investments in terms of hardware, software, and staff training. Integrating AI may disrupt existing clinical workflows, at least during the initial stages of implementation. Healthcare providers must adapt to new processes, potentially causing temporary inefficiencies. The integration of AI may require custom development and integration with existing clinical systems, which can be technically complex and costly. This complexity can hinder widespread adoption.

#### 4.2.4 Physician’s and Patient’s Acceptance

No studies have been performed that show that the implementation of AI indeed leads to higher quality of care, lower healthcare costs, or improved patient outcomes. Patient privacy and compliance with regulations regarding patient data are critical considerations. Physicians are not yet prepared for the implementation of AI in the daily clinical setting. Another potential risk is the impact of AI on the doctor-patient relationship. Patients may become concerned if they feel that their care is being determined solely by a machine rather than by a human healthcare professional who can provide a personalized approach [193]. Physicians are accustomed to making critical decisions based on their clinical expertise. Trusting AI-driven recommendations and giving up some autonomy in decision-making can be a significant barrier. Physicians are responsible for protecting patient data, and they may have concerns about how AI systems handle sensitive health information, particularly in the context of data breaches and privacy violations. Many AI algorithms, particularly DL models, can be complex “black boxes” that are challenging to interpret. Some physicians may be skeptical of AI systems due to a lack of comprehensive clinical validation and a limited understanding of the rigorous testing processes that underpin these technologies. Smaller healthcare facilities and those in resource-limited settings may lack the necessary infrastructure and funding to adopt and support AI technologies. Patients may not fully understand the capabilities and limitations of AI, leading to unrealistic expectations or skepticism about its effectiveness. Patients are understandably concerned about the security and privacy of their health data when AI systems are involved. Patients who lack access to technology or have limited digital literacy may face barriers to engaging with AI-driven healthcare services.

#### 4.2.5 Potential Challenges

The ML model’s performance decreases as datasets include data from classes with various probabilities of occurrence. Adversarial attacks compromise the reliability and robustness of DL methods and their safe application in medicine. They encompass mildly altered images, which resemble original images, but they are maliciously designed to confuse pre-trained models. This can lead to a completely different prediction for the image that the neural network analyses [21]. The model obtained by a DL method can be likened to a black box, which can be viewed in terms of its inputs and outputs without knowledge of its internal workings. Currently, no DL network can adequately explain its decision-making process, so acceptance in the medical industry is also a problem [194]. AI poses a novel challenge because the algorithms often require access to large quantities of patient data and may use the data in different ways over time [195]. Deploying and managing AI models across a large number of edge devices that bring computation and data storage closer to the data source, or the “edge” of the network, where the data are generated, can be challenging because it requires efficient model distribution, updates, and monitoring [196]. While wearable technology has the potential to transform cardiovascular care, it can increase health inequity, owing to the need for adequate digital affordability and literacy associated with wearable technology. The model’s predictive power starts decreasing with increasing dimensional complexity, often referred to as the ‘Hughes phenomenon’. Multi-dimensional data, when fed, leads to a lower degree of bias but a high variability. This leads to higher accuracy when tested internally but a dismal external performance [197].

Another issue that has raised ethical concerns for AI implementation in clinical practice is physicians’ professional liability in the case of an incorrect decision. Consequently, a complication or medical malpractice may be further perplexed since both healthcare professionals and AI developers are involved. AI-ECG algorithms may also be prone to overfitting, resulting in poor performance in test sets with limited generalizability [198].

## CONCLUSION

The incorporation of AI into cardiology is set to fundamentally transform the discipline, particularly influencing the diagnosis and management of arrhythmia. This innovative synergy between technology and medicine has the potential to reshape the landscape of cardiac care. AI-powered tools and algorithms are already being used to improve the accuracy and efficiency of ECG analysis, identify patients at high risk of arrhythmias, and guide interventional therapies. The progression of AI in cardiology has paved the way for a future where predictive algorithms, smart devices, and real-time monitoring systems are instrumental in improving patient outcomes. The quality and availability of data, regulatory frameworks, ethical considerations, and the integration of AI into existing clinical workflows are hurdles that require careful navigation. There is a need to integrate AI tools and algorithms into existing clinical workflows and to ensure physician and patient acceptance. Ethical considerations, such as ensuring patient data privacy and navigating the potential for algorithmic bias, must also be carefully managed. There is a pressing need for clear regulatory frameworks that define standards for AI deployment in healthcare settings, ensuring safety, efficacy, and transparency. As AI continues to evolve, it is critical to anticipate and mitigate its potential risks, ensuring that its integration serves to complement, rather than replace, human expertise. In the future, AI is poised to play an even greater role in cardiology, enabling personalized and predictive care for patients with arrhythmias.

## Figures and Tables

**Fig. (1):**
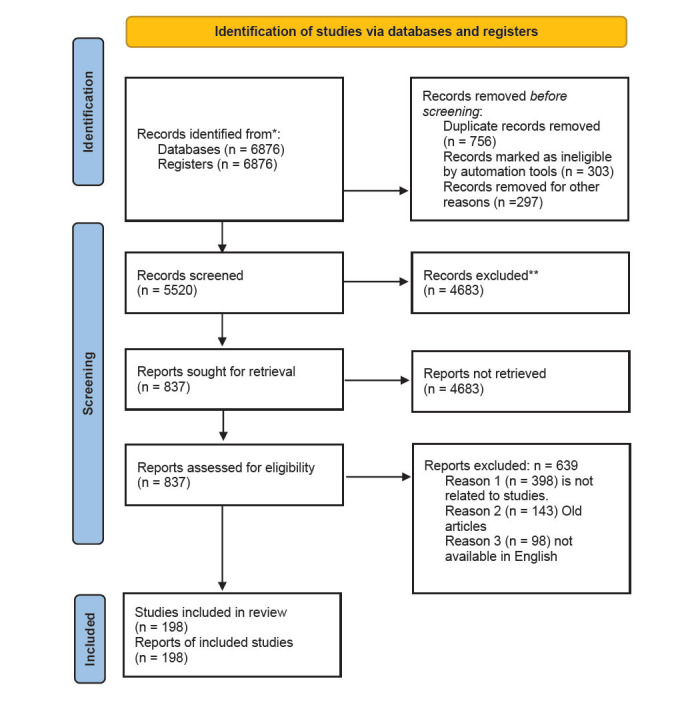
Schematic diagram for the selection of study articles.

**Fig. (2):**
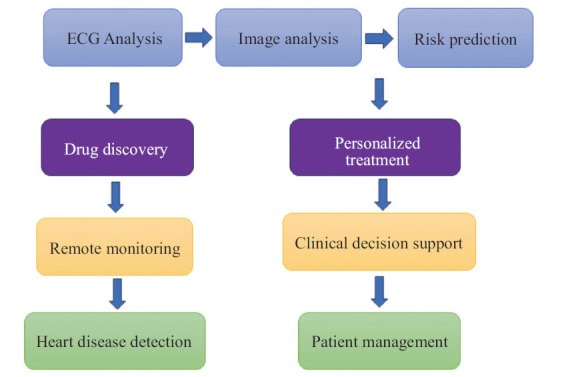
Clinical decision support system (CDSS).

**Fig. (3):**
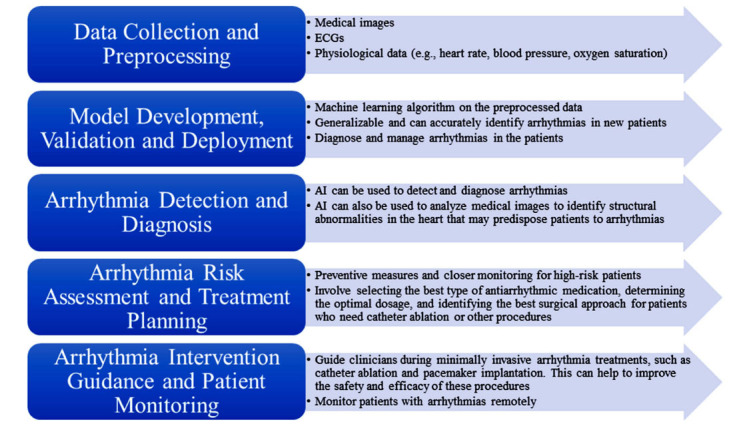
Artificial intelligence in cardiology.

**Fig. (4):**
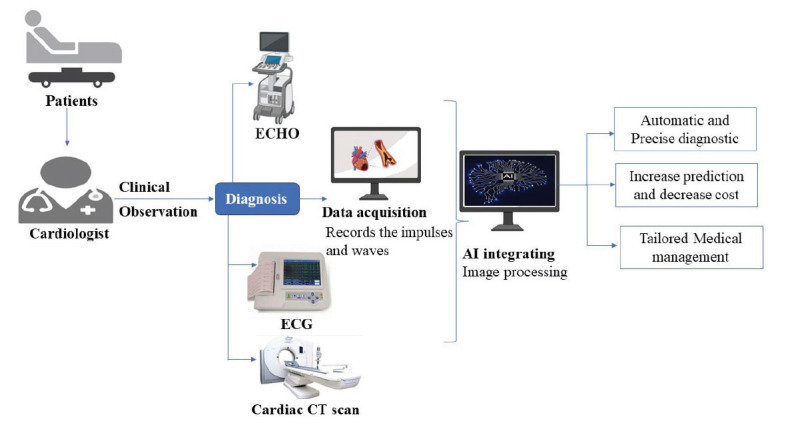
Swift image analysis by artificial intelligence.

**Fig. (5):**
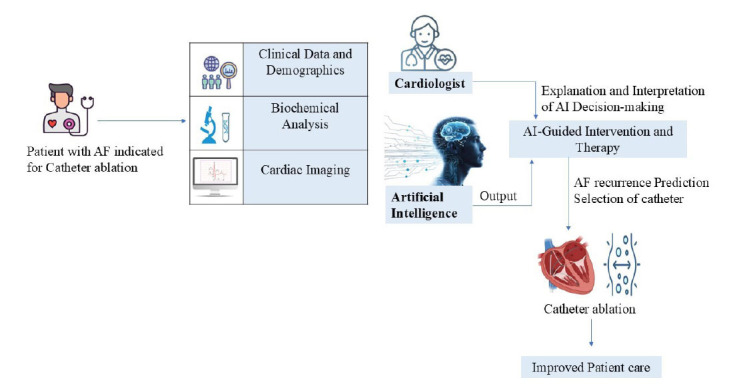
Catheter ablation procedure.

**Table 1:** Key clinical trials and studies using AI in cardiology.

**Trial Name**	**Objectives**	**Patient Population**	**AI Techniques Employed**	**Major Findings**	**References**
**Arrhythmias**					
Explainable DNN for LTVA in DCM	To detect DCM patients at risk of LTVA using an inherently explainable DNN.	695 DCM patients	Variational Autoencoder DNN	Factors F8, F15, F25, F27, and F32 are significantly associated with LTVA, mainly driven by P-wave abnormalities. VAE network combined with an interpretable Cox regression can distinguish patients at risk of LTVA.	[36]
Immune Cells in Atherosclerotic Plaques Study	Investigate immune cell role and diagnostic biomarkers in plaque stability	Stable and unstable carotid atherosclerotic plaques	CIBERSORT, Machine Learning (LASSO, Random Forest)	Unstable plaques had increased M0, M1, and M2 macrophages, decreased CD8+ T cells and NK cells. Diagnostic markers: CD68, PAM, IGFBP6. M1 macrophages drive instability. Predicted therapies: insulin, nivolumab, indomethacin, α-mangostin.	[37]
ML for Heart Failure Phenogrouping Study	Categorize HF patients for CRT response using ML	1106 HF patients from MADIT-CRT	Unsupervised ML (Multiple Kernel Learning, K-means clustering)	Identified four phylogroups with distinct clinical characteristics and treatment responses. Two phenogroups showed significantly better CRT-D treatment effects on the primary outcome. Proof-of-concept for optimizing therapy responders using clinical and imaging data.	[38]
Automated LVEF Measurement with DPS-Net	Evaluate a DL algorithm for automated LVEF measurement using 2DE images	34,090 2DE frames from 340 patients	Deep Learning (DPS-Net), U-Net, Biplane Simpson's method	DPS-Net achieved high performance in LV segmentation and LVEF measurement for various heart disease phenotypes and ultrasound systems. Outperformed EchoNet-dynamic in LV segmentation. High diagnostic performance (AUC 0.948-0.974) for normal hearts and different heart disease phenotypes. DPS-Net is adaptable to various echocardiographic systems.	[39]
**Echocardiography**					
AI-ECG AF Risk Study	Predict AF risk with AI using 12-lead ECGs	45,770 patients at Massachusetts General Hospital	Convolutional Neural Network (ECG-AI)	ECG-AI and CHARGE-AF models provide complementary and efficient AF risk quantification.	[40]
Automated Echocardiogram Interpretation Study	Create a fully automated echocardiogram analysis pipeline	14,035 echocardiograms	Convolutional Neural Networks	Accurate view identification (96% accuracy), segmentation, and quantification of cardiac structure and function. Detection of hypertrophic cardiomyopathy, cardiac amyloidosis, and pulmonary arterial hypertension (C statistics: 0.93, 0.87, 0.85). Potential for scalable analysis of millions of echocardiograms for patient tracking.	[41]
ECG Rhythm Classification Study	Develop an ensemble of RNNs for heart rhythm classification in ECG records	12,186 single-lead ECG recordings	Recurrent Neural Networks	Achieved state-of-the-art classification performance with an average F1 score of 0.79 for distinguishing normal sinus rhythms, atrial fibrillation, other arrhythmias, and noisy signals. Enhanced interpretability through an attention mechanism.	[42]
Cardiac Motion Analysis Study	Use AI to predict survival in cardiac patients	302 patients with cardiac MR images	Fully convolutional network, supervised denoising autoencoder, Cox partial likelihood loss	AI model significantly outperformed human benchmarks (Harrell's C-index: 0.75 *vs.* 0.59) in survival prediction using cardiac motion data. Demonstrates efficient survival prediction using high-dimensional medical images.	[43]
ASteRisk Clinical Risk Prediction Model Study	Predict AS risk using ML with ECG.	1,130 patients with AS	Machine learning (Bootstrap Lasso Regression, Ridge Logistic Regression)	Identified 9 key features for predicting all-cause mortality or AVR for up to 5 years. The model performed well, especially for years 2-5 and in patients with LGAS. Model's generality was tested on an independent dataset (AUC 0.78). The ASteRisk score is publicly available.	[44]
AI-Enabled QTc Estimation Study	Develop an AI algorithm to estimate QTc using 12-lead ECGs and a mobile ECG (mECG) device	>1.6 million 12-lead ECGs from 538,200 patients	Deep neural network - AI	DNN accurately predicts QTc values from 12-lead ECGs. QTc values from mECG closely match 12-lead ECGs. High accuracy (AUC 0.97, sensitivity 80.0%, specificity 94.4%) in detecting QTc ≥500 ms with mECG. Suggests cost-effective screening for long QT syndrome using mECG.	[45]
**Valvular Heart Disease**					
Valvular Heart Disease Study	Develop ECG AI to detect AS, AR, and MR	77,163 patients who underwent ECG within 1 year before echocardiography (AS, AR, or MR)	Deep Learning	AI achieved high accuracy in detecting AS (AU-ROC: 0.88), AR (AU-ROC: 0.77), MR (AU-ROC: 0.83), and any of AS, AR, or MR (AU-ROC: 0.84; sensitivity 78%, specificity 73%).	[46]

**Abbreviations:** AF: Atrial fibrillation, AR: aortic regurgitation, AS: aortic stenosis, AVR: aortic valve replacement, DCM: dilated cardiomyopathy, DNN: deep neural network, MR: mitral regurgitation, LTVA: life-threatening ventricular arrhythmia, LVEF: left ventricle ejection fraction.

**Table 2:** Benefits of AI in improving the accuracy and efficiency of diagnosis.

**Author Name**	**Study Title**	**AI Technique Used**	**Statistical Results & Conclusion**	**References**
Tang *et al.*	AI-based Early Detection of CAD *via* ECG	Convolutional Neural Network	The CAD detection model achieved an AUC of 0.75 with 70.0% accuracy, demonstrating efficiency in noninvasive CAD detection solely through ECG.	[166]
Papandrianos and Papageorgiou	Deep Learning in SPECT MPI for CAD Diagnosis	Deep Learning and CNN	Utilizing SPECT MPI scans, a CNN model achieved an overall classification accuracy of 93.47% ± 2.81% and an AUC score of 0.936 in distinguishing ischemia or infarction from healthy patients.	[167]
Abubaker and Babayiğit	Deep Learning for Predicting Cardiac Abnormalities	Transfer Learning, CNN	The CNN model performed excellently, achieving 98.23% accuracy, 98.22% recall, 98.31% precision, and 98.21% F1 score in predicting four cardiac abnormalities. It also achieved the highest score of 99.79% when employed for feature extraction with the Naïve Bayes algorithm.	[168]
Liastuti *et al.*	AI in Diagnosing Left Heart Failure through Echocardiography	Various Machine Learning Algorithms (including CNN)	Accuracy varies from 57% to 99.3%; AI complements clinicians for better and faster diagnosis of left heart failure through echocardiography.	[169]
Kashou *et al.*	AI-ECG Algorithm for Comprehensive 12-Lead ECG Interpretation	Convolutional Neural Network (CNN)	Area under the ROC curve of ≥0.98 for 62 of the 66 codes; Sensitivity ≥95% for all codes; AI-ECG demonstrates high diagnostic performance compared to reference cardiologist interpretation.	[170]
Kulkarni and Amin	ANN Models for Predicting Adverse Outcomes after PCI	Artificial Neural Networks (ANN) - Multilayer Perceptron (MLP)	AKI: AUC 77.9%, Bleeding: AUC 86.5%, Death: AUC 90.3%, Any Adverse Outcome: AUC 80.6%, Unsatisfactory Prediction for Stroke (AUC 69.9%).	[171]
Chang Junior *et al.*	Mortality Prediction in CHD Patients Undergoing Cardiac Surgery	Multilayer Perceptron (MLP), Random Forest (RF), Extra Trees (ET), Stochastic Gradient Boosting (SGB), Ada Boost Classification (ABC), Bag Decision Trees (BDT)	RF achieved AUC of 0.902. Predictors: prior ICU admission, diagnostic group, height, hypoplastic left heart syndrome, body mass, arterial oxygen saturation, and pulmonary atresia (67.8% importance in RF). “Hospital death” rates higher in patients up to 66 cm height with BMI below 13.0; decreased with increased arterial oxygen saturation. Prior hospitalization associated with higher rates. Diagnoses groups align with international literature. Web app CgntSCORE for mortality prediction.	[172]
Choi *et al.*	AI-CDSS for Heart Failure Diagnosis	Hybrid Approach	Retrospective cohort (test dataset concordance rate: 98.3%). Concordance rates for heart failure with reduced EF, mid-range ejection fraction, preserved EF, and no heart failure: 100%, 100%, 99.6%, and 91.7%, respectively. Prospective pilot study (AI-CDSS *vs.* non-heart failure specialists): Concordance rate 98% *vs.* 76%. AI-CDSS showed high diagnostic accuracy for heart failure.	[173]
Betancur *et al.*	ML for MACE Prediction with SPECT MPI	Boosted Ensemble Algorithm	During 3.2 ± 0.6 years follow-up, 9.1% (239 patients) had MACE. ML-combined (clinical and imaging data) showed higher predictive accuracy than ML-imaging (AUC: 0.81 *vs.* 0.78; *p* < 0.01). ML-combined outperformed MD diagnosis, automated stress TPD, and automated ischemic TPD (AUC: 0.81 *vs.* 0.65 *vs.* 0.73 *vs.* 0.71; *p* < 0.01 for all). Risk reclassification for ML-combined *vs.* MD diagnosis was 26% (*p* < 0.001).	[174]

**Abbreviations:** ANN- Artificial neural networks; CDSS- clinical decision support system; AKI- acute kidney injury; AUC- appropriate use criteria; CAD- coronary artery disease; CHD- congenital heart defect; CNN- convolutional neural network; ECG- electrocardiogram; EF- ejection fraction; MACE- major adverse cardiovascular events; ML- machine learning; MLP- multilayer perceptron; ROC- receiver operating characteristic; TPD- total perfusion deficit.

## Data Availability

All data generated or analysed during this study are included in this published article.

## LIST OF ABBREVIATIONS

AF: Atrial Fibrillation
AI: Artificial Intelligence
AKI: Acute Kidney Injury
AMI: Acute Myocardial Infarction
ANN: Artificial Neural Networks
AR: Aortic Regurgitation
AS: Aortic Stenosis
AUC: Area Under the Curve
AVNRT: Atrioventricular Nodal Reentrant Tachycardia
AVR: Aortic Valve Replacement
BrS: Brugada Syndrome
CA: Catheter Ablation
CAC: Coronary Artery Calcium
CAD: Coronary Artery Disease
CDSS: Clinical Decision Support System
CHD: Congenital Heart Defect
CHF: Chronic Heart Failure
CI: Cardiac Imaging
CIED: Cardiac Implantable Electronic Devices
CMR: Cardiac Magnetic Resonance
CNN: Convolutional Neural Network
CRT: Cardiac Resynchronization Therapy
CS: Coronary Stenosiss
CVD: Cardiovascular Disease
DCM: Dilated Cardiomyopathy
DL: Deep Learning
DNN: Deep Neural Network
ECG: Electrocardiograms
EF: Ejection Fractions
EHR: Electronic Health Record
HCM: Hypertrophic Cardiomyopathy
HRV: Heart Rate Variability
ICD: Implantable Cardioverter-Defibrillators
LQTS: Long QT Syndrome
LTVA: Life-Threatening Ventricular Arrhythmia
LV: Left Ventricular
LVEF: Left Ventricle Ejection Fraction
MACE: Major Adverse Cardiovascular Events
ML: Machine Learning
MLP: Multilayer Perceptron
MPI: Myocardial Perfusion Imaging
MR: Mitral Regurgitation
MRI: Magnetic Resonance Imaging
PCE: Pooled Cohort equation
PCI: Percutaneous Coronary Interventions
PET: Positron Emission Tomography
PPG: Photoplethysmography
PRISMA: Preferred Reporting Items for Systematic Reviews and Meta-Analyses
RCT: Randomized Controlled Trial
ROC: Receiver Operating Characteristic
RWMA: Regional Wall Motion Abnormalities
SCA: Sudden Cardiac Arrest
SCD: Sudden Cardiac Death
SCDA: Specific Risks of Arrhythmic Sudden Cardiac Death
SE: Stress Echocardiography
SPECT: Single-Photon Emission Computed Tomography
SSCAR: Survival Study of Cardiac Arrhythmia Risk
STFT: Self-contained Short-Time Fourier Transform
SVM: Support Vector Machines
TPD: Total Perfusion Deficit
VF: Ventricular Fibrillation
VHD: Valvular Heart Disease
VT: Ventricular Tachycardia

## STANDARDS OF REPORTING

PRISMA guidelines and methodology were followed.

## SUPPLEMENTARY MATERIAL

PRISMA checklist is available as supplementary material on the publisher’s website along with the published article.
